# Resolving the positions of defects in superconducting quantum bits

**DOI:** 10.1038/s41598-020-59749-y

**Published:** 2020-02-20

**Authors:** Alexander Bilmes, Anthony Megrant, Paul Klimov, Georg Weiss, John M. Martinis, Alexey V. Ustinov, Jürgen Lisenfeld

**Affiliations:** 1 Physikalisches Institut, Karlsruhe Institute of Technology, Karlsruhe, 76131 Germany; 2 Google, Santa Barbara, California, 93117 USA; 3 National University of Science and Technology MISiS, Moscow, 119049 Russia; 4 Russian Quantum Center, Skolkovo, Moscow, 143025 Russia

**Keywords:** Quantum information, Qubits, Characterization and analytical techniques

## Abstract

Solid-state quantum coherent devices are quickly progressing. Superconducting circuits, for instance, have already been used to demonstrate prototype quantum processors comprising a few tens of quantum bits. This development also revealed that a major part of decoherence and energy loss in such devices originates from a bath of parasitic material defects. However, neither the microscopic structure of defects nor the mechanisms by which they emerge during sample fabrication are understood. Here, we present a technique to obtain information on locations of defects relative to the thin film edge of the qubit circuit. Resonance frequencies of defects are tuned by exposing the qubit sample to electric fields generated by electrodes surrounding the chip. By determining the defect’s coupling strength to each electrode and comparing it to a simulation of the field distribution, we obtain the probability at which location and at which interface the defect resides. This method is applicable to already existing samples of various qubit types, without further on-chip design changes. It provides a valuable tool for improving the material quality and nano-fabrication procedures towards more coherent quantum circuits.

## Introduction

Material defects have a manifold of microscopic origins such as impurities in solids or adsorbates hosted on surfaces^1^. Their detrimental role was identified already in first experiments with superconducting quantum bits (qubits)^2^. Strong interaction with a long-known defect type, charged two-level systems (TLS)^3,4^, residing in the tunnel barrier of a qubit’s Josephson junction gives rise to avoided level crossings and resonant energy absorption^5^. This form of dielectric loss could be mitigated by reducing the amount of lossy dielectrics, e.g. by incorporating smaller Josephson junctions^6^ and by avoiding insulating layers. Another strategy is to enlarge the footprint of device capacitors in order to dilute the electric field induced by the qubit, which excites defects by coupling to their electric dipole moments^7,8^. As a consequence of significantly enhanced coherence times, qubits became sensitive also to weakly coupling defects residing on the surfaces and interfaces of circuit electrodes^9^. Since these are limiting the performance of state-of-the-art circuits^10–12^, further progress towards scaled-up quantum processors requires strong efforts to prevent the appearance of defects, e.g. by using better materials, improved fabrication procedures^13–16^, and surface treatment to avoid contamination and parasitic adsorbates^17,18^. This endeavor needs to be guided by careful analysis of defect properties such as densities, electric dipole moments, and positions, in order to identify and improve the manufacturing steps that reduce defect formation, and to analyze the microscopic structure of defects.

In this Letter, we present a method to extract information on the spatial positions of defects at the profile of the film edge in a qubit circuit. For doing this, we exploit the tunability of a charged defect’s resonance frequency *ω* by an electric field **E**,

1 $$\omega =\sqrt{{\varDelta }^{2}+{\varepsilon }^{2}}/\hslash ,\,\,\varepsilon ={\varepsilon }_{i}+2{\bf{p}}{\bf{E}},$$

where *ℏ* is the reduced Planck constant, and **p** is the defect’s electric dipole moment. The offset energy *ε*_*i*_ is given by local strain and electric fields from surrounding atoms, and *Δ* is the defect’s (constant) tunneling energy^3,19^. In our experiment, **E** is composed of electric fields **E**_t_ and **E**_b_ generated by two gate electrodes placed above (t) and below (b) the sample chip, respectively. The qubit is used to monitor the defect’s resonance frequency and their responses to DC voltages *V*_t_ and *V*_b_ applied to the respective top and bottom electrode. Comparing the response to the spatial variation of the applied electric fields obtained from finite element simulations, the position **x** of a defect can be deduced by solving the equation

2 $${\gamma }_{{\rm{t}}}{V}_{{\rm{t}}}/{\gamma }_{{\rm{b}}}{V}_{{\rm{b}}}={\bf{p}}{{\bf{E}}}_{{\rm{t}}}({V}_{{\rm{t}}},{\bf{x}})/{\bf{p}}{{\bf{E}}}_{{\rm{b}}}({V}_{{\rm{b}}},{\bf{x}}).$$

Here, *γ*_t_ and *γ*_b_ are the defect’s tunability coefficients by the respective top and bottom fields, which are obtained by fitting the measured resonance frequency dependence of each defect

3 $$\omega =\sqrt{{\Delta }^{2}+{\left({\varepsilon }_{{\rm{i}}}+{\gamma }_{{\rm{t}}}{V}_{{\rm{t}}}+{\gamma }_{{\rm{b}}}{V}_{{\rm{b}}}\right)}^{2}}/\hslash $$

derived from Eq. (1) using the identities 2**pE**_t_ = *γ*_t_*V*_t_ and 2**pE**_b_ = *γ*_b_*V*_b_. Since DC electric fields approach metallic electrodes always perpendicular to their surface, Eq. (2) can be reduced by dropping the dipole moment projections onto each field, and regarding the absolute field values. Thus, at metal film interfaces the defect locations are deduced from the simplified equation *γ*_t_/*γ*_b_ = |**E**_t_|/|**E**_b_|. At the substrate-vacuum interface however, the applied fields are not necessarily parallel, which requires accounting for the defect’s dipole moment orientation, as described below.

## Experiment

**Figure 1:**
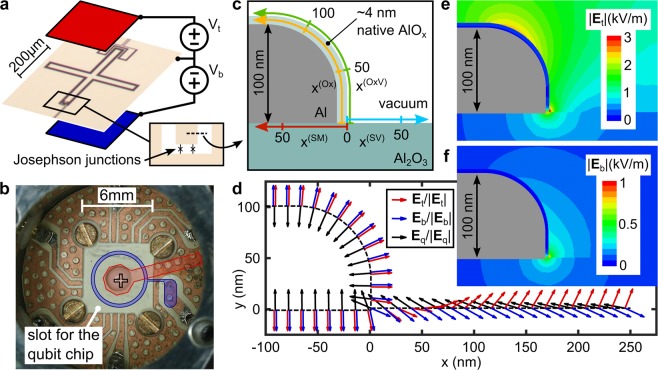
Experimental setup for E-field tuning of defects and simulation results. (**a**) Qubit picture with sketched top (red) and bottom (blue) electrodes which are controlled by two independent voltage sources referenced to the on-chip ground plane. (**b**) Photograph of the opened sample housing without qubit chip, and illustration of the top (red) and annular bottom electrode (blue). The cross mark denotes the location of the investigated qubit. (**c**) Sketch of a cross-section through the substrate and the qubit electrode near the film edge, showing the interfaces of interest: the substrate-metal (SM) and substrate-vacuum (SV) interfaces, the native AlO_*x*_ layer (Ox), and the oxide-vacuum interface (OxV). Colored arrows define the coordinate systems along each interface, which have their origins close to the substrate-metal-vacuum edge. (**d**) Directions of the simulated DC fields **E**_t_ and **E**_b_ generated by top and bottom electrodes, and of the qubit’s plasma oscillation field **E**_q_. (**e**,**f**) Simulated electric field strengths for a voltage of − 0.5 V applied to either top or bottom electrodes.

We connect each of the two electrodes to a DC voltage source that is referenced to the common ground of the sample housing and the on-chip groundplane as sketched in Fig. 1a. A photograph of the opened aluminum sample housing is shown in Fig. 1b, where red and blue structures indicate the real dimensions of the DC electrodes. The top electrode consists of a copper/Kapton-foil sandwich glued to the lid of the sample housing. The bottom electrode is integrated in the PCB backplane. Its circular shape allows a piezo-mechanical transducer to exert mechanical force onto the center of the qubit chip, which allows one to tune defects by elastic strain^20,21^. In this work, the piezo is not used, and we refer to ref. ^22^ for a study comparing the defect response to mechanical strain and electric fields that was performed with the same setup. Further technical details of the sample housing are provided in Supp. Mat. 1.

The employed qubit sample is an aluminum-based transmon qubit^23^ consisting of a cross-shaped capacitor electrode that is connected to ground by a split Josephson junction^9^. The applied E-field is expected to be constant along the film edges of the qubit island and of the surrounding groundplane due to the qubit’s geometric symmetry and its central position relative to the electrodes (see Supp. Mat. 1). Accordingly, the problem to simulate the spatial dependence of the electric field can be limited to a 2-dimensional cross-section focusing on the film edge where the fields are strongest. This region is illustrated in Fig. 1c, labeling the interfaces of interest which are the substrate-vacuum (SV), and the three film interfaces: substrate-metal (SM) interface, the inside of the native aluminum oxide layer (Ox), and the oxide-vacuum interface (OxV). The colored arrows indicate the spatial coordinates along each interface that have their origins at the substrate-metal-vacuum edge. As confirmed by SEM investigation, the edge of the film is rounded because it was patterned using isotropic reactive ion etching.

The E-field distribution that results from simulations is shown in Figs. 1d–f. In Fig. 1d, the direction of applied fields and of the qubit’s AC electric field **E**_q_ at the different circuit interfaces are indicated by colored arrows. Figure 1e,f show the electric field strengths generated by applying a voltage of − 0.5 V to one of the electrodes. These show that the top and bottom fields are supposed to be focused in different regions, which is the key point to resolve locations of defects from their response to each gate electrode. Furthermore, the qubit AC field is concentrated in the same region as the DC fields, which implies that all defects residing at the investigated interfaces and detectable by the qubit couple to the global electrodes, as detailed in Supp. Mat.1.

**Figure 2:**
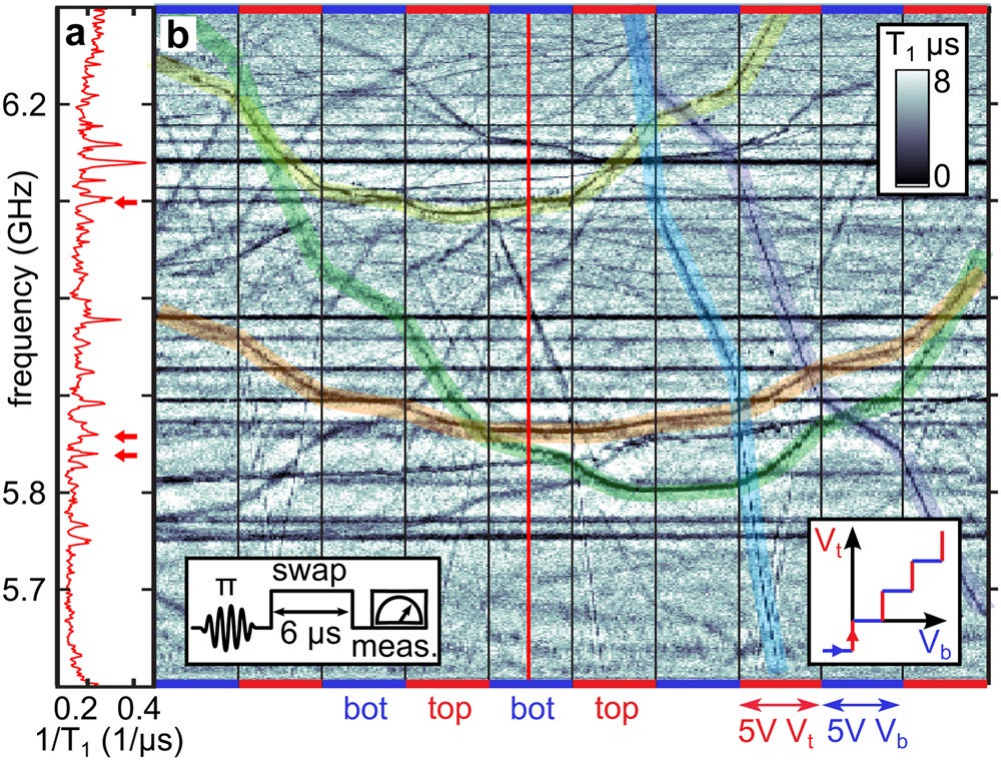
Tuning defects by electric fields. (**a**) Frequency-dependent energy relaxation rate 1∕*T*_1_ of the qubit, measured by a swap spectroscopy protocol sketched in the left inset of (**b**). Distinct peaks stem from resonant interaction with individual defects. (**b**) Dependence of defect resonance frequencies (dark traces) on applied voltage ramps alternating the top (red margins) and the bottom electrode (blue margins). The right inset contains the underlying ramp path in the voltage plane. Colored curves in the main figure highlight hyperbolic fits to Eq. (3) with asymptotic slopes *γ*_t/b_ which reflect the asymmetry tunability of defects by each electrode, characteristic of their position. The red vertical line indicates the exemplary trace shown in **(a)** where red arrows denote resonances of the highlighted defect traces. Horizontal black lines stem from defects hosted in the qubit’s stray Josephson junction as explained in a previous work^22^.

The resonance frequencies of defects are detected by qubit swap spectroscopy^5,20^ using the protocol shown in the left inset of Fig. 2b. This provides a measurement of the qubit’s energy relaxation rate 1/*T*_1_ whose frequency dependence displays Lorentzian peaks due to resonant interaction with defects, as visible in Fig. 2a. To obtain the defects’ response coefficients *γ*_t_ and *γ*_b_ required to solve Eq. (2), we alternate measurements where the voltage on either the top or the bottom electrodes is swept upwards. The right inset of Fig. 2b illustrates such a sweeping path in the *V*_**t**_ − *V*_**b**_ space, and the main figure shows resulting data. Segmented hyperbolic traces of individual defects exhibit unequal slopes in reaction to the two gates. According to Fig. 1e,f, these slopes reflect different local field strengths **E**_t_ and **E**_b_. A few exemplary fits to Eq. (3) are indicated by highlighted curves in Fig. 2b. Horizontal traces indicate defects which most probably reside inside the qubit’s stray Josephson junction where they do not experience any applied DC fields, as further detailed in the previously mentioned work^22^.

**Figure 3:**
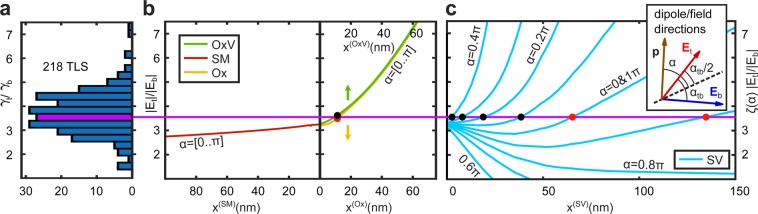
Deduction of defect positions. (**a**) Measured ratios *γ*_t_/*γ*_b_ of defect tunability coefficients by the top and bottom electrodes. (**b**) Ratio of the simulated electric field strengths |**E**_t_|/|**E**_b_| along the different interfaces as indicated by the axes labels. Since the applied DC fields are parallel at these interfaces, the field ratio does not depend on *α* which defines the defect’s dipole moment orientation (see inset of (**c**)). (**c**) Right side of Eq. (2) for some *α* values, where $\zeta (\alpha ,{\bf{x}})\equiv \cos (\alpha -{\alpha }_{{\bf{tb}}}({\bf{x}})/2)/\cos (\alpha +{\alpha }_{{\bf{tb}}}({\bf{x}})/2)$ is the ratio of dipole moment projections onto the fields, and *α*_**tb**_ is the angle between **E**_t_ and **E**_b_. The horizontal violet line through (**b**,**c**) provides graphical solutions of Eq. 2 for possible positions of an exemplary defect (black dots), of which those requiring nonphysically large electric dipole moments >10 Debye ≈ 2*e* are discarded (red points).

A distribution of measured *γ*_t_/*γ*_b_ ratios is plotted in Fig. 3a, comprising data from 218 field-dependent defects that could be detected with this qubit in a frequency range of 0.7 GHz (between 5.6 GHz and 6.3 GHz) and an applied voltage range of *V*_t_, *V*_b_ ∈ [−100. . 100] V. As expected, all defects are more strongly tuned by the top electrode that induces a larger field at the qubit position, due to its geometry (cf. Fig. 1b).

## Results

As stated before, defect locations at film interfaces (SM, Ox, and OxV) are given by solutions of *γ*_t_/*γ*_b_ = |**E**_t_|/|**E**_b_|. The ratio of simulated electric field strengths is shown in Fig. 3b along the respective coordinates defined in Fig. 1c. The horizontal violet line through Fig. 3b provides graphical solutions of Eq. (2) for an exemplary defect with measured ratio *γ*_t_/*γ*_b_ ≈ 3.5. The two solutions at the Ox and OxV interfaces are indicated by red and black dots, respectively, and correspond to a defect’s distance of ~15 nm from the substrate-metal-vacuum edge. Since the applied fields are parallel at film interfaces, the solutions are degenerate in the dipole moment orientation *α* ∈ [0. . *π*] defined in the inset of Fig. 3c. At the SV interface, the tunability ratio depends in addition on *α*, as visualized in Fig. 3c where the right term of Eq. (2) is plotted for some *α* values. Hereby, $\zeta (\alpha ,{\bf{x}})\equiv \cos (\alpha -{\alpha }_{{\rm{tb}}}({\bf{x}})/2)$/$\cos (\alpha +{\alpha }_{{\rm{tb}}}({\bf{x}})/2)$ is the ratio of dipole moment projections on the fields, and *α*_tb_ is the angle between **E**_t_ and **E**_b_. The red and black dots indicate anticipated locations of the exemplary defect at the SV interface.

The defect’s electric dipole moment component *p*_∥_ parallel to the applied fields is calculated from each solution **x** and a corresponding field strength **E**_**t**/**b**_(**x**), as reported in Supp. Mat. 1. Inside the oxide layer (Ox), electric fields are reduced by about a factor of 10 due to the materials’ permittivity. A possibility for the exemplary defect (*γ*_t_ = 102 *ℏ*MHz/V, *γ*_b_ = 29 *ℏ*MHz/V) to reside at this interface would require an electric dipole moment of *p*_∥_ ≈ 30 Debye, which is unrealistic. Therefore, we discard in our analysis all solutions that imply dipole moments *p*_∥_ > 10 Debye ≈ 2*e* which is a maximum imaginable number in solids^24^. The cutoff value is discussed in Supp. Mat. 3. In Fig. 3b,c, valid and truncated solutions from the current example are indicated by black and red dots, respectively. Regarding possible locations at the SV interface, only angles *α* ∈ [0.1. . 0.4] π lead to reasonable dipole moment sizes for this defect, corresponding to distances *x*^SV^ ∈ [40. . 5] nm from the substrate-metal-vacuum edge. The probability for a given defect to reside at a particular interface is defined by the range of allowed *α*. Here, *α* spans over 0.4 π at the SV, and over π at the OxV interfaces, resulting in *P*_SV_ = 0.4*π*/(*π* + 0.4*π*) ≈ 0.29 and *P*_OxV_ = *π*/(*π* + 0.4*π*) ≈ 0.71, respectively. This calculation is repeated for each field-tunable defect using hundreds of interpolated *α* values to increase the probability precision.

**Figure 4:**
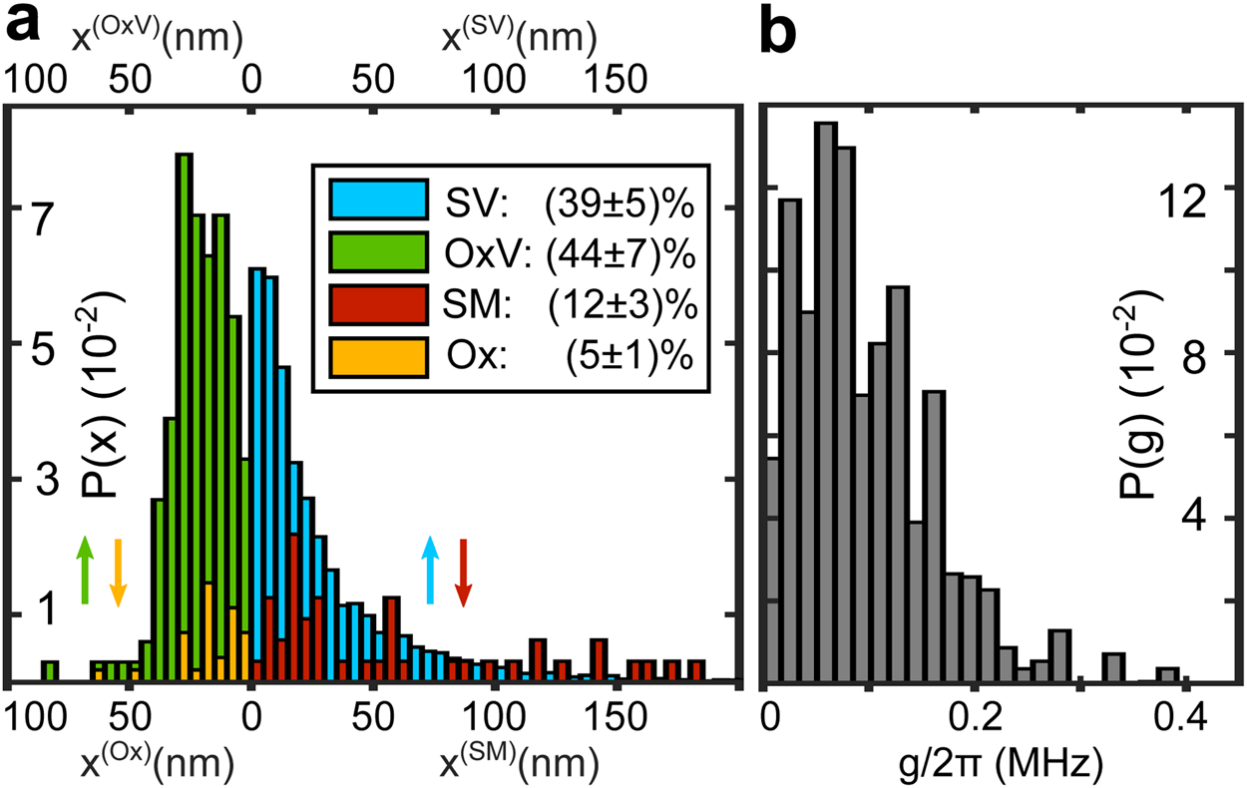
Defect positions and coupling strengths. (**a**) Histograms of deduced defect locations, whose relative weights reveal the probability of finding a field-tunable defect at the respective interface (see legend). The probability errors are deduced from the estimation error of the electrode distances to the qubit film as detailed in Sup. 3. The interface to air (OxV, SV) hosts most detectable defects, possibly due to fabrication contaminants and adsorbates. Further, detected defects are concentrated within 50 nm from the substrate-metal-vacuum edge since the qubit fields are focused in this region. (**b**) Distribution of extracted defect-qubit coupling strengths *g*, which is in good agreement to direct measurements using the same sample^22^.

The main result of this work is presented in Fig. 4a, showing the histogram of extracted positions of field-tunable defects weighted by the average probability to reside at a certain interface (colors). Most of such defects are located within a distance of about 50 nm to the substrate-metal-vacuum edge (*x* = 0). This is expected since the qubit AC-electric field is mainly concentrated in this region. More distant defects couple too weakly to be detected with the given qubit coherence time. With highest probability, defects reside at open surfaces of the sample, i.e. at the OxV and SV interfaces. Due to the reduced strength of applied fields inside the native oxide, most solutions at the Ox interface resulted in unrealistic dipole moment sizes, and were discarded. The SV histogram appears particularly broad and smoothed due to tracing out the dipole moment orientation *α* which is assumed isotropic.

Once the defect positions are identified, we can calculate their coupling strengths to the qubit *g* = **pE**_q_(**x**) from the deduced dipole moment projections on the applied fields and the simulated qubit AC field **E**_q_. Figure 4b shows the resulting distribution of *g*, which is very similar to an independent and direct measurement of the coupling strengths reported previously^22^.

We note that our analysis does not resolve defect locations along the qubit film edge, especially it disregards the possibility that defects reside on the narrow electrodes of the qubit’s split Josephson junction. However, this region may be particularly critical because of the concentrated qubit field, and the additional lithography steps required to deposit sub-micron junctions, which may promote defect formation by substrate surface amorphisation and processing residuals^15^. Nevertheless, we expect that the field strength ratio remains unchanged at the edges of junction-forming films so that the analysis remains valid in this region.

Overall, in this sample, we detected on average 16 defects per GHz hosted in the Josephson junctions, and 26 field-tunable defects per GHz at any applied electric field. Further 5 field-tunable defects per GHz could not be located since some defects appeared only in one data segment (red and blue framed windows in Fig. 2b). This effect can be minimized by choosing sufficiently narrow segments. Assuming an equal distribution of field-tunable defects along the 3mm-long edge of the qubit film, we obtain a density of ≈10 defects per (GHz ⋅ mm). We note that the sample was stored for several years while it was covered by photoresist. Incorporation of resist atoms and residuals due to inadequate cleaning may explain the degradation of qubit coherence caused by an increased number of surface defects detected in this work.

## Conclusions

The demonstrated technique to determine the position of defects on the surface of a quantum circuit provides a viable tool to verify the material quality and to optimize micro-fabrication steps. Our technique requires only few externally placed gate electrodes and is thus applicable directly to existing qubit chips. Since the junction’s tunnel barrier is free of applied DC electric fields, junction defects can be easily identified by zero field-tunability. While this technique presumably applies to all qubit geometries built in a coplanar architecture, the analysis of our sample is certainly least time-consuming due to its geometric symmetry. This method can be further improved by performing independent measurements of the qubit-defect coupling strengths *g* and thus their effective dipole moment sizes^22^, hereby reducing uncertainties concerning the interface at which a defect resides.

We have found that 46% of all defects reside on the surface of the qubit sample, and 10% are hosted inside the native oxide or at the metal-substrate interface. The location of another 10% could not be resolved, and 34% of defects reside in the tunnel barrier of the qubit’s stray Josephson junctions. Hereby, the amount of defects in the weak junctions is negligible. While the redundant stray junctions can be simply shorted^15^, qubit surfaces remain another dominant and inherent source of dielectric losses. Decreasing this loss requires rigorous studies of fabrication processes and surface treatments during or after sample fabrication in order to improve the surface quality. The reported technique can be used to examine dielectric losses at film edges in suspended or trenched qubit samples, where electric fields can be more diluted while, on the other side, a larger surface for hosting adsorbates is available. In future experiments, additional on-chip gate electrodes can provide enhanced spatial resolution helping to distinguish defects in the immediate vicinity of the tunnel junctions.

## Methods

The supplementary material contains details on the experimental setup, simulations of the electric fields, plots of complete measured data sets, and the analysis method with error estimation. Further details can be found in the PhD thesis of A.B.^25^.

## Supplementary information

Supplementary Information.

## Data Availability

The supplementary material contains details on the experimental setup, simulations of the electric fields, plots of complete measured data sets, and the analysis method with error estimation. The datasets generated and analysed during the current study are available from AB on request.
