# Publisher Correction: Resolving the positions of defects in superconducting quantum bits

**DOI:** 10.1038/s41598-020-61788-4

**Published:** 2020-03-10

**Authors:** Alexander Bilmes, Anthony Megrant, Paul Klimov, Georg Weiss, John M. Martinis, Alexey V. Ustinov, Jürgen Lisenfeld

**Affiliations:** 1 Physikalisches Institut, Karlsruhe Institute of Technology, Karlsruhe, 76131 Germany; 2 Google, Santa Barbara, California, 93117 USA; 3 National University of Science and Technology MISiS, Moscow, 119049 Russia; 4 Russian Quantum Center, Skolkovo, Moscow, 143025 Russia

Correction to: *Scientific Reports* 10.1038/s41598-020-59749-y, published online 20 February 2020

This Article contains an error in the Figure 3 legend and the second paragraph of the Results.

“The horizontal violet line through (**b,c**) provides graphical solutions of Eq. 2 for possible positions of an exemplary defect (black dots), of which those requiring nonphysically large electric dipole moments >10 Debye ≈ 2e are discarded (red points).”

should read:

“The horizontal violet line through (**b,c**) provides graphical solutions of Eq. 2 for possible positions of an exemplary defect (black dots), of which those requiring nonphysically large electric dipole moments >10 Debye ≈ 2eÅ are discarded (red points).”

“Therefore, we discard in our analysis all solutions that imply dipole moments *p*_∥_ > 10 Debye ≈ 2e which is a maximum imaginable number in solids^24^.”

should read:

“Therefore, we discard in our analysis all solutions that imply dipole moments *p*_∥_ > 10 Debye ≈ 2eÅ which is a maximum imaginable number in solids^24^.”

Furthermore, this Article contains an error in the conclusions.

“The demonstrated technique to determine the position of defects on the surface of a quantum circuit provides a viable tool to verify the material quality and to optimize micro-fabrication steps.”

should read:

“The demonstrated technique to determine the position of defects on the surface of a quantum circuit provides a viable tool to verify the material quality and to optimize nano-fabrication steps.”

