# Supplementary material for: Resolving the positions of defects in superconducting quantum bits
Source: Sci Rep. 2020 Feb 20;10:3090. doi: 10.1038/s41598-020-59749-y (PMC7033136; doi:10.1038/s41598-020-59749-y)
Supplement: Supplementary file 1 — Supplementary Information. [file 41598_2020_59749_MOESM1_ESM.pdf]

Supplementary material for Resolving the positions of defects in superconducting quantum bits

Alexander Bilmes*,[1] Anthony Megrant,[2] Paul Klimov,[2] Georg Weiss,[1]
John M. Martinis,[2] Alexey V. Ustinov,[1, 3, 4] and Jürgen Lisenfeld[1]
[1]*Physikalisches Institut, Karlsruhe Institute of Technology, 76131 Karlsruhe, Germany*
[2]*Google, Santa Barbara, California 93117, USA*
[3]*National University of Science and Technology MISiS, Moscow 119049, Russia*
[4]*Russian Quantum Center, Skolkovo, Moscow 143025, Russia*
(Dated: February 7, 2020)
(*alexander.bilmes@kit.edu)

1. Experimental setup, qubit sample, and E-field simulations

The sample measured in this work was fabricated by Barends et al. as described in Ref. [1]. This chip contained three uncoupled transmon qubits in 'Xmon' geometry, each consisting of a cross-shaped capacitor electrode connected to ground via two Josephson junctions in parallel. Between sample fabrication and our measurements, about four years have past during which the sample was covered with photoresist which might not have been completely removed prior to our measurements. We thus note that incorporation of contaminants from the resist and its residuals may enhance the number of surface defects detected in our experiments.

We use a standard scheme for qubit readout based on detection of the state-dependent dispersive frequency shift of a readout resonator coupled to each qubit as described in the supplementary material to Ref. [2]. The electric field is generated by two electrodes located above and below the qubit chip, which are both connected to independent voltage sources that are referenced to the cryostat body and on-chip grounds. Figure **S1a** shows a sketch of the sample housing's cross-section, and Figure **S1b** shows the three-dimensional (3D) model used for simulations of the generated electric field. The dimensions of the employed DC-electrodes are shown in Fig. **S1c**. Fields are simulated with finite element solver ANSYS Maxwell 2015 (release 16.2.0). Once the electric fields are characterized in a full 3D-simulation, we employ the spatially reduced 2D model illustrated in Fig. **S1d** in which the real electrodes are replaced by effective ones and a much denser meshing grid is used. Fig. **S1e** shows dimensions of the modelled film edge, and Figure **S2** shows the magnitudes of electric fields along the different circuit interfaces obtained from this simulation.

Although we acquired data from all three qubits, in our article we only discuss results obtained on the qubit that is located in the centre of the sample chip (Xmon 2), because only for this qubit the applied electric field is sufficiently homogeneous along the edge of the qubit island and of the surrounding ground plane. Figure **S3** illustrates this spatial dependence of the ratio of electric fields generated by top and bottom electrodes. In future experiments, the field homogeneity can be enhanced by using electrodes that are larger than the chip, allowing one to measure many qubit samples in the same cool-down.

The simulations show that the electric field induced by qubit oscillations is concentrated at film edges and decays as $\sim 1/\sqrt{x}$, where x is the distance to the film edge. Hereby, a defect having an electric dipole moment of $10\,\mathrm{D}$ (maximum imaginable value in solids) and residing at $x > 200\,\mathrm{nm}$ would couple to the qubit by $g/2\pi < 0.05\,\mathrm{MHz}$, which is about the typical detection sensitivity of the sample. Thus, $200\,\mathrm{nm}$ is the maximum distance to the edge where defects are still detectable. Further, simulations show that within such a distance, and on both sides of the film, the dc-electric field induced by each separate global electrode exceeds $130\,\mathrm{V/m}$ per volt applied (the maximum applied voltage is $\pm 100\,\mathrm{V}$). By this minimum field, the asymmetry energy of a defect having a small parallel electric dipole moment component of 1 Debye would be tuned by 130 MHz. Thus, we can be sure that every detectable defect is also clearly tunable by any of the two employed global electrodes.

2. Defect spectroscopy

Defects are detected by recording the frequency-dependence of the qubit energy relaxation rate which displays Lorentzian peaks due to dissipation from resonant defect interaction [3, 4]. To minimize the measurement time,

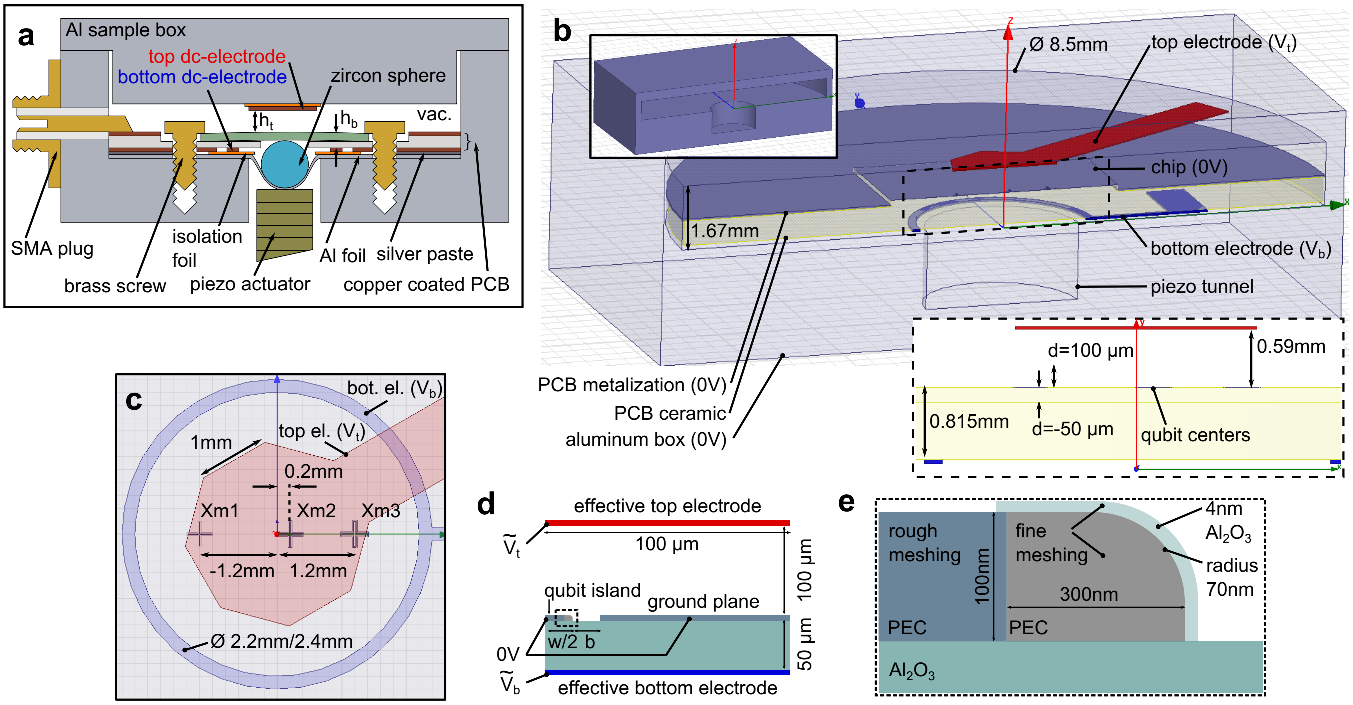

Figure S1. **Setup and simulations for electric field tuning of defects. a** Sketched cross-section of the sample housing. The piezo actuator is not necessary for this experiment. However, it was useful to obtain independent data sets in the same cool down, as explained in Fig. **S6**. **b** Model for coarse field simulations created with ANSYS Maxwell. The main plot shows a cut-away of the sample cavity which contains a printed circuit board (PCB, light yellow) carrying planar microwave lines* to which the sample chip is wire-bonded. The sample housing contains a circular hole in its center (piezo tunnel) allowing a piezo actuator* to exert force onto the qubit chip in order to tune defects by mechanical strain as employed in Ref. [2]. Therefore, the bottom electrode below the chip has a circular shape. The top electrode consists of a copper foil/Kapton foil sandwich that is glued to the lid of the sample housing. (*) not contained in the simulation model. **c** shows the exact dimensions of the DC-electrodes from a top view. The bottom electrode is ring-shaped to leave space for the zircon sphere which mediates the elastic strain applied to the chip using the piezo actuator. The grey crosses indicate the positions of the 3 Xmon qubits on the chip. In this work, we only discuss data obtained on the centre qubit (Xm2) for which the electric fields are constant along the edge of the qubit islands. **d** Reduced 2D-model to enhance the precision of field simulations. The electrodes are modelled by effective parallel plates located 50 and 100 μm below and above the chip, respectively. These are biased at reduced voltages $\widetilde{V}_{\rm t/b}$ so that the electric fields have equal strengths as those generated by the real electrodes, which we find from the full 3D simulation. The qubit island potential is set to ground due to the transmon regime of the qubit. **e** Magnified view of the film edge profile. Only a small margin of the edge cross-section is fine resolved (maximum mesh width 0.1 nm) since the qubit fields are concentrated at the film edge, and defects residing further away are not detectable by the qubit. The rest of the model is automatically meshed with a maximum mesh width of 250 nm. The aluminum film is modelled as a perfect conductor (PEC), and the amorphous native oxide on top of the aluminum is represented by a 4 nm thick sapphire film.

we employ a swap spectroscopy protocol and deduce the energy relaxation rate from a single measurement at each frequency and additional reference measurements. More details on the employed methods can be found in the supplementary material to Ref. [2].

3. Error estimation

Electrode distance

We estimate that the largest error in the deduced defect locations stems from uncertainties in the vertical distances $h_{\rm t}$ and $h_{\rm b}$ between the qubit and the top and bottom electrodes, respectively. Deviations in $h_{\rm b}$ may result from uneven machining of the PCB ceramic, while $h_{\rm t}$ depends on the thicknesses of insulation foils underneath the qubit chip and between the top electrode and sample housing. To estimate their influence on the results, we repeat our analysis for systematically varied electrode distances within a range of $\pm 50\,\mu$m (the estimated maximum error) around their nominal values $h_{\rm t} = 590\,\mu$m and $h_{\rm b} = 815\,\mu$m. This results in small differences for deduced defect locations and

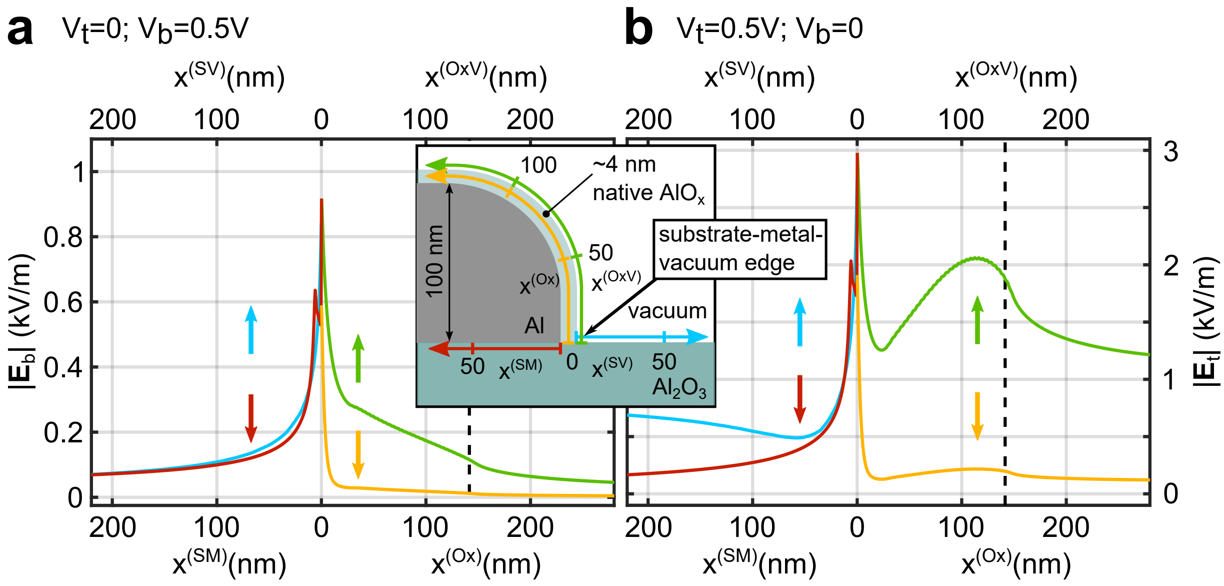

Figure S2. **Results from E-field simulations.** Strength of the electric field generated by applying a voltage of 0.5 V to either **a** the bottom electrode or **b** the top electrode, along the substrate-vacuum (SV) interface (blue), the oxide surface (OxV, green), the substrate-metal interface (SM, red), and the inside of the oxide layer (Ox, yellow). The spacial axes along the interfaces are indicated by arrows of corresponding colors. The inset illustrates the coordinates along the different interfaces, which have their origins at the substrate-metal-vacuum edge.

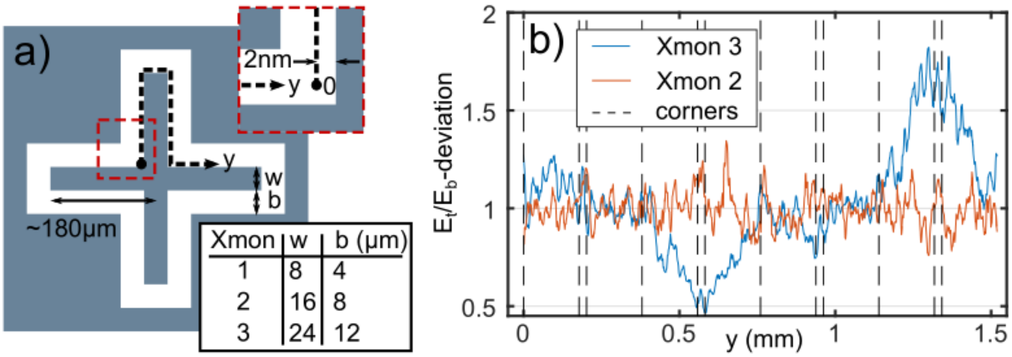

Figure S3. **Homogeneity of the electric field.** **a** Illustration of the Xmon qubit geometry. The dashed line shows the path along which the homogeneity of the electric field is verified. **b** Deviation of the electric field strength ratio $|\boldsymbol{E}_\mathrm{t}/\boldsymbol{E}_\mathrm{b}|$ relative to the mean value, plotted along the dashed path in **a**. The electric field was simulated in the 3D-model using a coarse resolution to save calculation time, which explains the plot roughness. For the center qubit (Xmon2, red line), the field ratio is constant along the qubit edge, in contrast to the lateral qubits. The dashed vertical lines indicate the positions of corners of the cross-shaped qubit electrode.

interface participations whose arithmetic mean values and standard deviations are quoted in the legend of Fig. 4 **a** of the main text. While the numbers presented in Fig. 4 **a** have been deduced from a sweep using a step of 10 μm, an exemplary result using a coarse variation step of 25 μm is presented in Fig. **S4** to give an impression how the analysis outcome behaves under small distance variations. In each subplot, the underlying $(h_\mathrm{t}, h_\mathrm{b})$ combination is indicated while the empty frames denote combinations outside of the allowed limit of $\pm 50\,\mu$m. Further, the distance variations obey the fact that h_t depends on changes in h_b due to the sample holder geometry (see Fig. **S1a**), but not vice versa. For example, if we choose h_t to be shortened by 50 μm and h_b by 25 μm, the resulting distances will be $(h_\mathrm{t} - 50\,\mu\mathrm{m} + 25\,\mu\mathrm{m}, h_\mathrm{b} - 25\,\mu\mathrm{m}) = (565, 790)\,\mu\mathrm{m}$.

Film edge shape

Deviations in the shape of the qubit electrode from the assumed rounded profile, e.g. due to fabrication imperfections or local film corrosion, will result in local electric field strengths that differ from the simulation results. However,

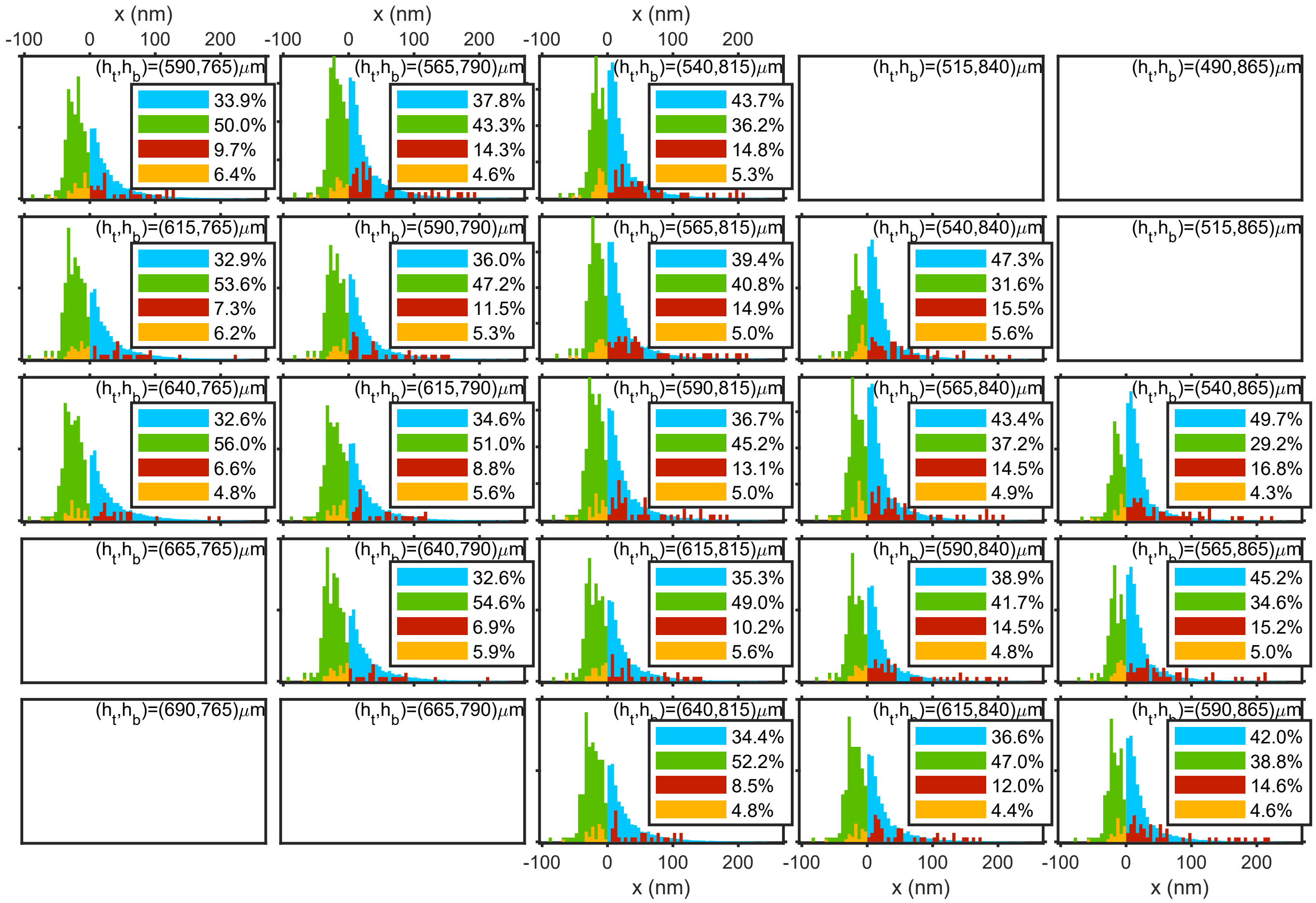

Figure S4. **Error estimation.** Defect locations at different interfaces (colors) as deduced from analysis runs for varying distances h_{t} and h_{b} between the qubit and the top and bottom electrodes around their nominal values $h_{\mathrm{t}} = 590\,\mu\mathrm{m}$ and $h_{\mathrm{b}} = 815\,\mu\mathrm{m}$, and with a variation step of $25\,\mu\mathrm{m}$. The legends indicate the participation of the given interface to the total amount of detected field-tunable defects. The interface participations and errors quoted in Fig. 4 **a** of the main text result from the arithmetic mean and standard deviation of equivalent results obtained with a smaller variation step of $10\,\mu\mathrm{m}$.

since our analysis is based on the comparison of the strengths of E-fields induced by the top and bottom electrodes, which presumably are equally affected by film imperfections, we expect that the analysis remains valid.

Cutoff value

To discuss the 10 D cutoff value used in this work to truncate TLS location solutions, we have repeated the analysis for varying cutoffs. Figure **S5 a** shows that a larger cut-off value leads to a larger participation of the Ox interface, which is expected since this interface normally (when the 10 D cutoff is applied) contains most truncated location solutions. For cutoffs below 10 D (see **b**), the histograms become narrower around $x \approx 0$ (substrate-metal-vacuum edge), which is expected due to strongest qubit fields in this region. Moreover, smaller cutoffs lead to increased participation of the SV-interface. The reason stems from the method to calculate the probability for a single TLS to reside at a given interface, explained in the main text: TLS locations at film interfaces (OxV, SM, Ox) are degenerate in the TLS orientation and thus they are usually stronger weighted than the orientation-specific solutions at SV-interfaces. On the other hand, in contrast to film interfaces, solutions at SV-interfaces exist for every analyzed TLS even for very small cutoffs. Hence, if all film interface solutions of a TLS happen to be discarded due to a small cutoff value, the SV solutions are maximally weighted which leads to the peak at $x \approx 0$ in the SV histograms shown in this figure. To conclude, the 10 D cutoff is physically motivated: We consider 10 D as a largest reasonable dipole moment size in solids, and there is no reason to use larger cutoffs. However, smaller cutoffs lead to loss of reasonable solutions and to an unrealistic discontinuity of the location histogram at $x \approx 0$.

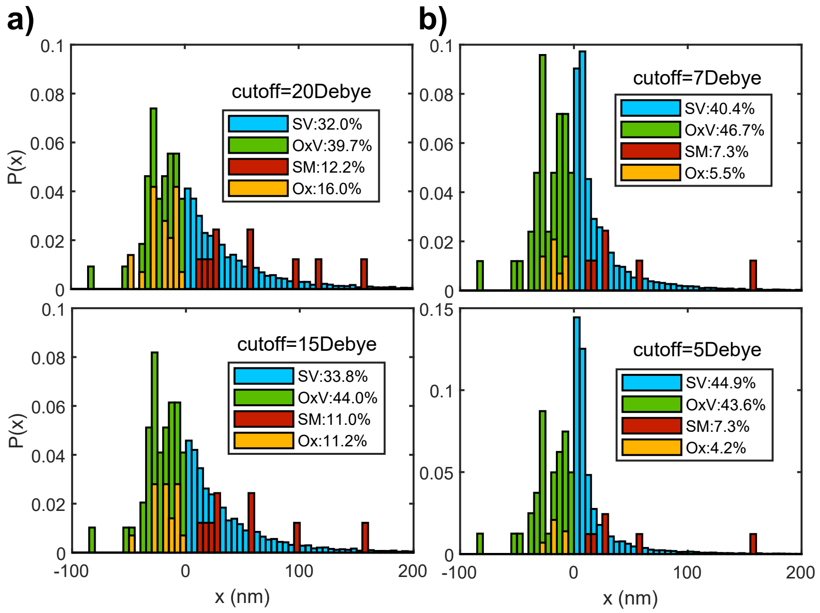

Figure S5. TLS location histograms and interface contributions (legends) deduced for cutoffs **a** beyond and **b** below the 10 D cutoff used in this work and by which the TLS locations are truncated.

4. Additional data

Figure **S6a** shows three data sets of defect tunabilities by top and bottom electrodes γ_t and γ_b, respectively, plotted against the defect's tunability ratios γ_t/γ_b, recorded with Xmon 2 in two cryogenic runs. Fig. **S6b** contains dipole moment components deduced from each defect location (cf. Fig. 3) at the film-interfaces (legend). All unrealistic solutions implying a dipole moment larger than 10 Debye are discarded (red line). The data points at tunability ratios below ~ 2.5 are missing here since the electric fields at the SM interface were not simulated beyond the distance of 220 nm from the substrate-metal-vacuum edge. An extrapolation however shows that the electric field at larger distances is so weak that the extracted dipole moments are far above the cutoff value.

5. Further details

The here discussed work was part of the PhD studies of Alexander Bilmes at Karlsruhe Institute of Technology (KIT). Further details on the experimental setup, electric field simulations, and data acquisition can be found in his thesis [5].

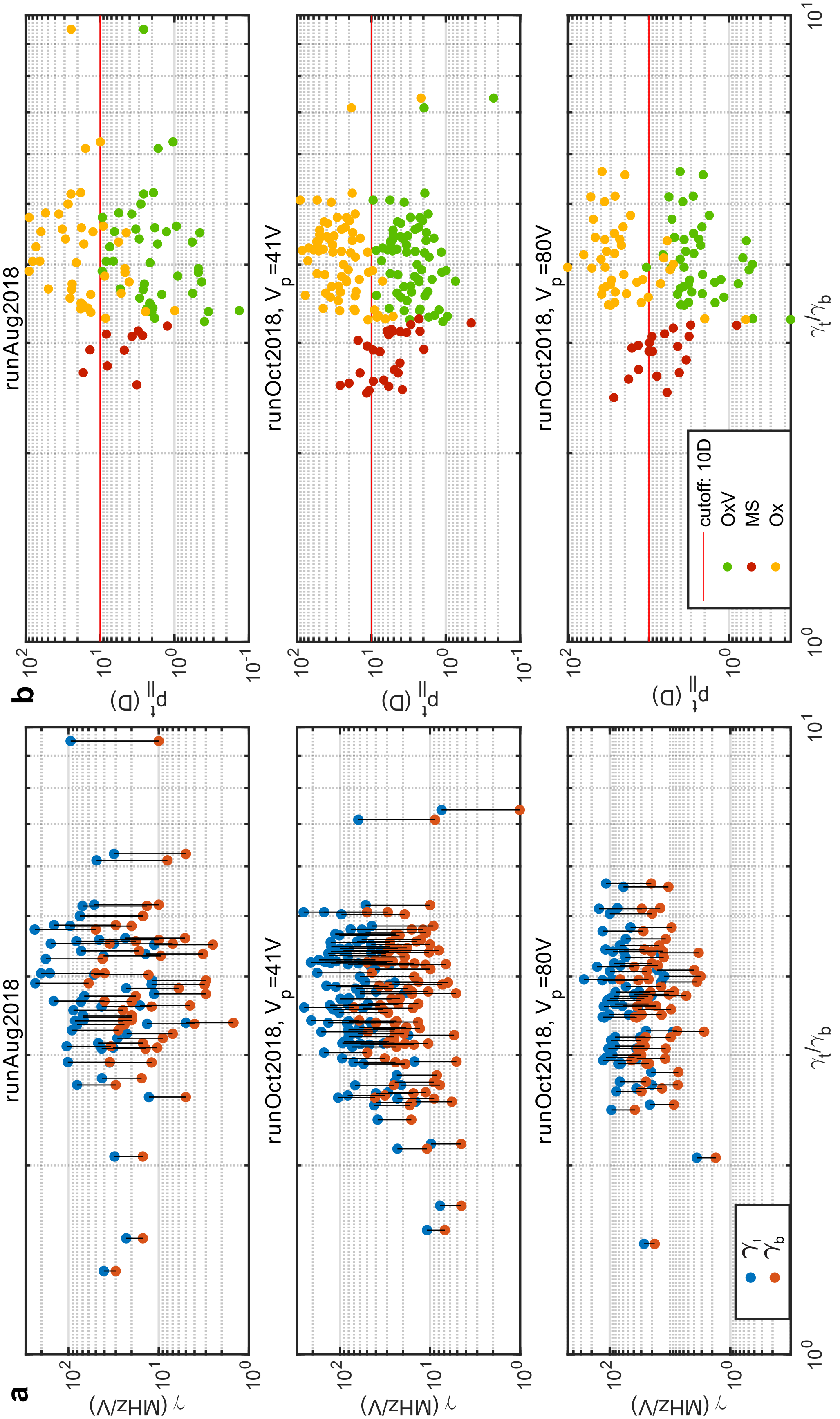

Figure S6. **Defect tunabilities and dipole moments a** Measured defect tunabilities by top and bottom electrodes γ_{t} and γ_{b}, respectively, plotted as a function of the tunability ratio $\gamma_{\mathrm{t}}/\gamma_{\mathrm{b}}$. From the cryogenic run from October 2018, two data sets exist that were recorded at very different elastic strain applied to the sample chip by a piezo actuator (control voltage V_{p}), which results in two different set of defects investigated. **b** Dipole moment components deduced from each defect location and the local electric field. In total, locations of 218 defects were analyzed.

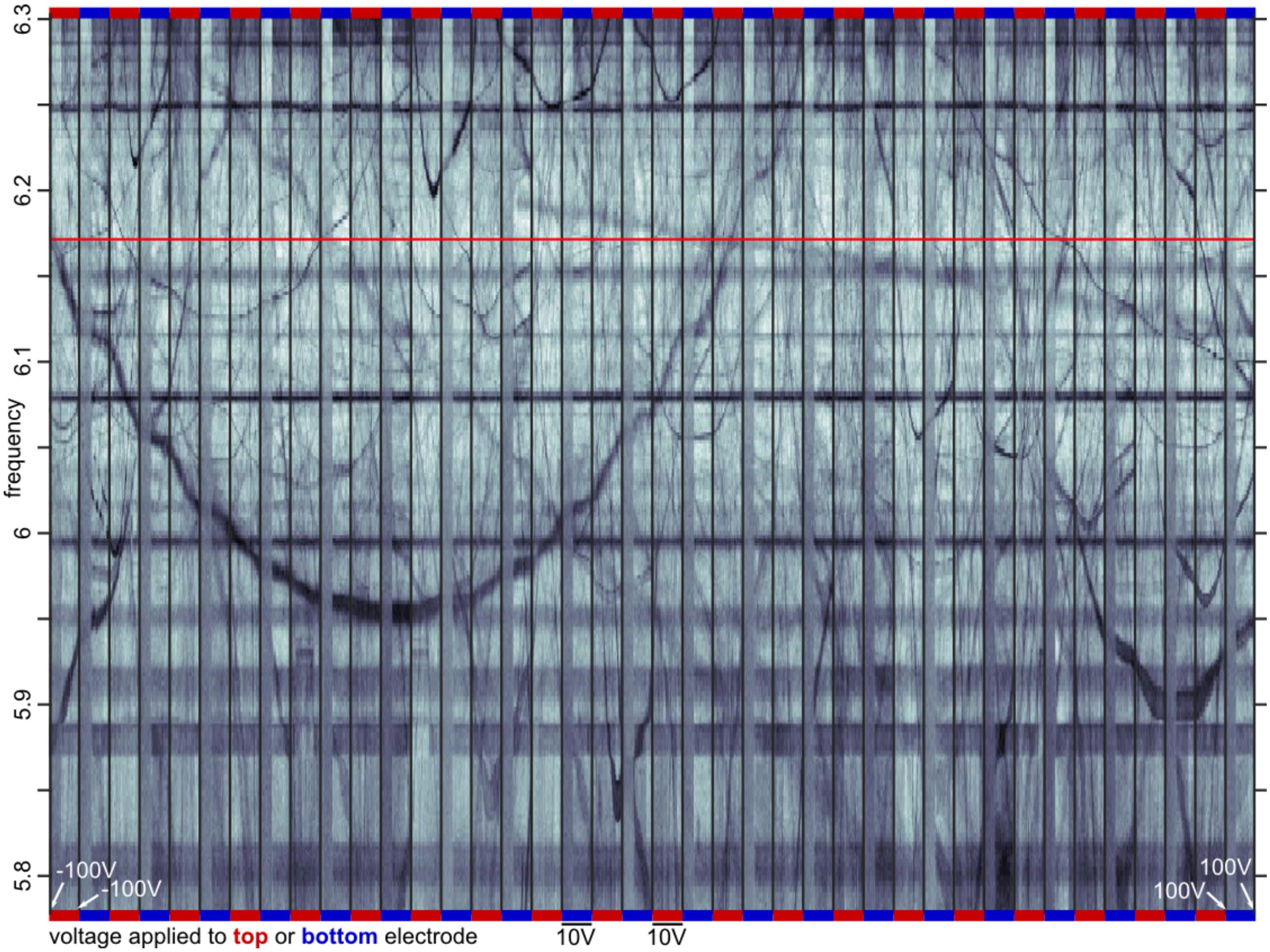

Figure S7. **Resonances of defects** (dark traces) recorded using swap spectroscopy while sweeping either the voltage applied to the top electrode (red margins) or bottom electrode (blue margins). Each segment spans a range of 10 V, the total range was -100 to +100 V at a step size of 0.14V, the frequency resolution was 3 MHz, and the total measurement duration was 26 hours. The red line indicates the qubit resonance frequency at zero applied magnetic flux. The qubit T_1 time was 8.3 μs and the swap duration was 6 μs.

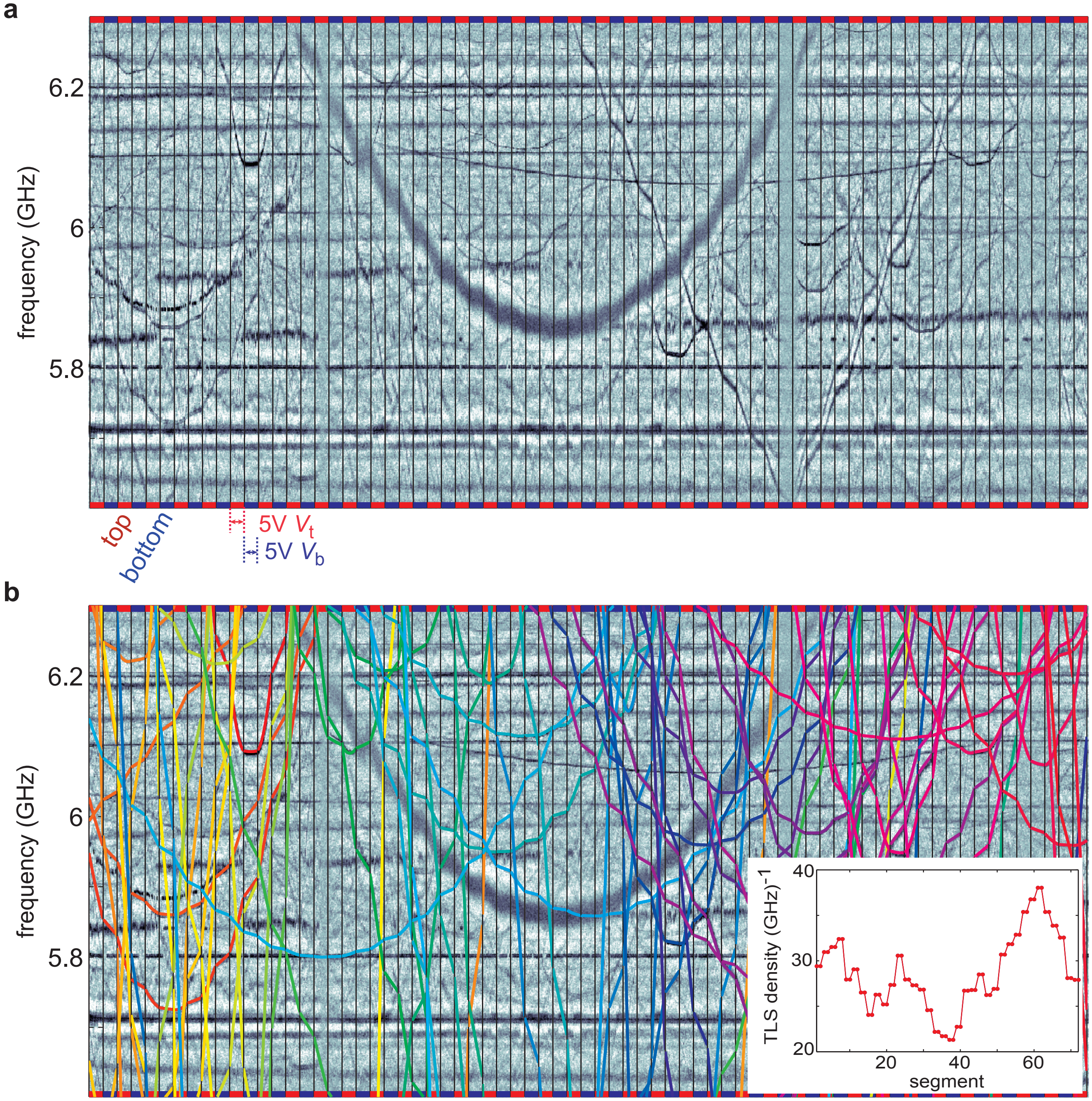

Figure S8. **Resonances of defects (dark traces)**. **a** Alternating sweeps of the voltage applied to the top electrode (red margins) and bottom electrode (blue margins). Each segment spans a range of 5 V, the total range was -80V to +100V at a step size of 0.14V, the frequency resolution was 1.5 MHz, and the total measurement duration was 59 hours. **b** Same data with superimposed fits to segmented hyperbolas (coloured lines). The inset shows the number of detected and fitted defect resonances per GHz in each segment, on average 29 defects/GHz, in total 99 defects.

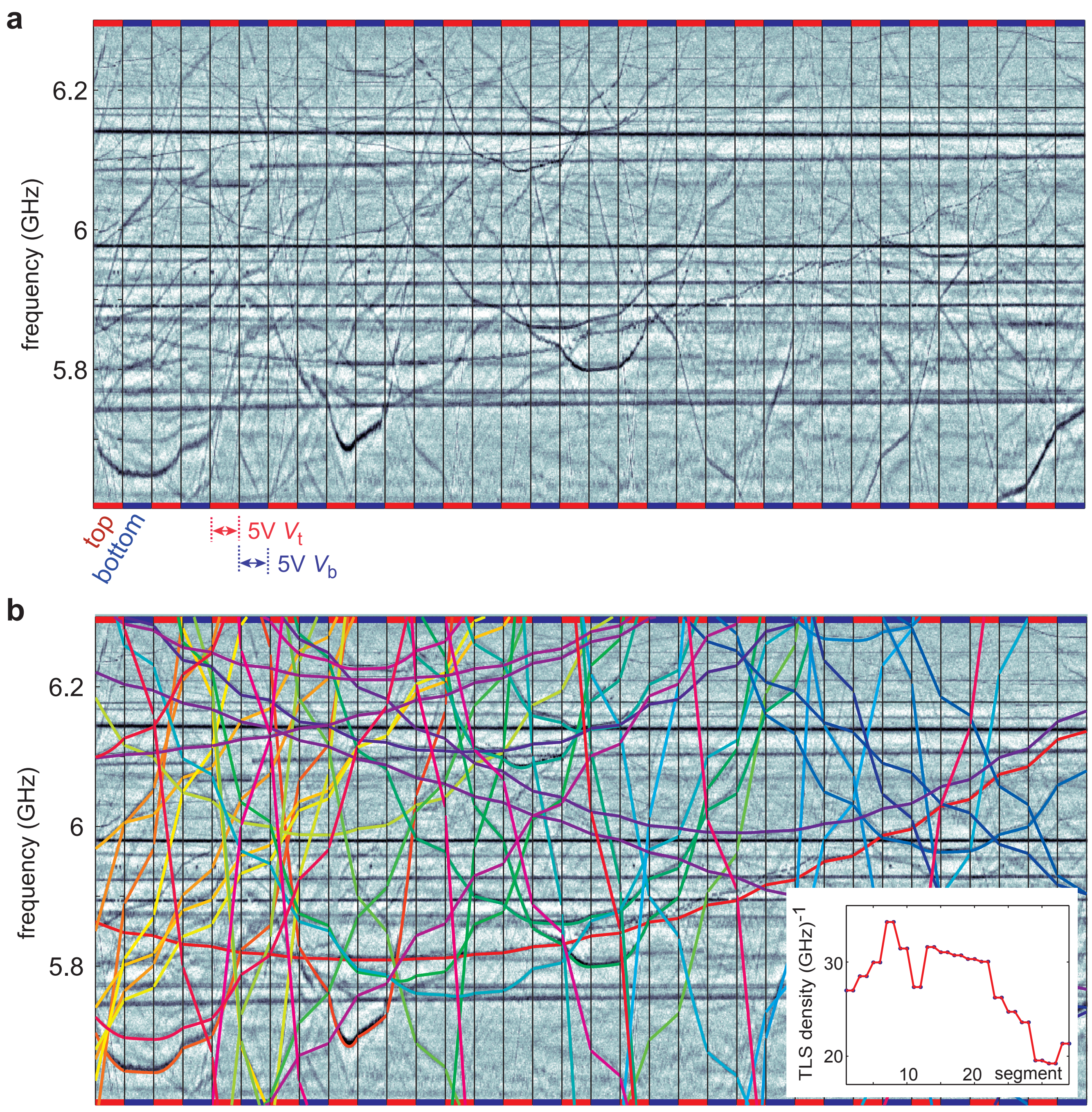

Figure S9. **Resonances of defects (dark traces)**. Same cool-down as in Fig. **S8**, but taken at a significantly different mechanical strain so that different defects are observed. **a** Alternating sweeps of the voltage applied to the top electrode (red margins) and bottom electrode (blue margins). Each segment spans a range of 5 V, the total range was 100 to 15 V at a step size of 0.14V, frequency resolution was 1.5 MHz, the total measurement duration was 23 hours. **b** Same data with superimposed fits to segmented hyperbolas (coloured lines). The inset shows the number of detected and fitted defect resonances per GHz in each segment, on average 27 defects/GHz, in total 63 defects.

[1] Barends, R. *et al.* Coherent josephson qubit suitable for scalable quantum integrated circuits. *Phys. Rev. Lett.* **111**, 080502 (2013). URL https://link.aps.org/doi/10.1103/PhysRevLett.111.080502.
[2] Lisenfeld, J. *et al.* Electric field spectroscopy of material defects in transmon qubits. *npj Quantum Information* **5**, 105 (2019).
[3] Cooper, K. B. *et al.* Observation of quantum oscillations between a josephson phase qubit and a microscopic resonator using fast readout. *Phys. Rev. Lett.* **93**, 180401 (2004). URL https://link.aps.org/doi/10.1103/PhysRevLett.93.180401.
[4] Lisenfeld, J. *et al.* Observation of directly interacting coherent two-level systems in an amorphous material. *Nat Commun* **6** (2015). URL https://doi.org/10.1038/ncomms7182.
[5] Bilmes, A. *Resolving locations of defects in superconducting transmon qubits.* dissertation, Karlsruhe Institute of Technology (KIT) (2019). URL http://dx.doi.org/10.5445/KSP/1000097557.
